# Prevention of sudden cardiac death in patients with chronic kidney disease

**DOI:** 10.1186/1471-2369-13-162

**Published:** 2012-12-03

**Authors:** Beata Franczyk-Skóra, Anna Gluba, Maciej Banach, Dariusz Kozłowski, Jolanta Małyszko, Jacek Rysz

**Affiliations:** 1 Department of Nephrology, Hypertension and Family Medicine, Medical University of Lodz, Lodz, Poland; 2 Department of Hypertension, WAM University Hospital in Lodz, Medical University of Lodz, Zeromskiego 113, Lodz, 90-549, Poland; 3 Department of Cardiology and Electrotherapy, Second Chair of Cardiology, Medical University of Gdansk, Gdansk, Poland; 4 Department of Nephrology and Transplantology, Medical University, Bialystok, Poland

**Keywords:** Arrhythmias, Chronic kidney disease, Renal failure, Sudden cardiac death

## Abstract

Cardiovascular deaths account for about 40% of all deaths of patients with chronic kidney disease (CKD), particularly those on dialysis, while sudden cardiac death (SCD) might be responsible for as many as 60% of SCD in patients undergoing dialysis. Studies have demonstrated a number of factors occurring in hemodialysis (HD) that could lead to cardiac arrhythmias. Patients with CKD undergoing HD are at high risk of ventricular arrhythmia and SCD since changes associated with renal failure and hemodialysis-related disorders overlap. Antiarrhythmic therapy is much more difficult in patients with CKD, but the general principles are similar to those in patients with normal renal function - at first, the cause of arrhythmias should be found and eliminated. Also the choice of therapy is narrowed due to the altered pharmacokinetics of many drugs resulting from renal failure, neurotoxicity of certain drugs and their complex interactions. Cardiac pacing in elderly patients is a common method of treatment. Assessment of patients’ prognosis is important when deciding whether to implant complex devices. There are reports concerning greater risk of surgical complications, which depends also on the extent of the surgical site. The decision concerning implantation of a pacing system in patients with CKD should be made on the basis of individual assessment of the patient.

## Introduction

Cardiovascular diseases (CVD) are the cause of about 50% of deaths in Europe [1,2]. Mortality of patients with chronic kidney disease (CKD) is associated with ischemic heart disease, myocardial infarction (MI) and sudden cardiac death (SCD) [3,4]. CKD is an independent risk factor for coronary artery disease [5]. Nowadays, a growing number of people require renal replacement therapy. In 2003, in Poland, 296 people per million were on renal replacement therapy and 135 people/million had functioning renal grafts [6]. Today the main causes of CKD are the following: diabetic nephropathy, chronic glomerulonephritis and hypertensive nephropathy [4,5].

In CKD patients common factors associated with accelerated atherosclerosis such as hyperlipidemia, hypertension, diabetes, acid–base balance and calcium phosphate metabolism, anemia, high prevalence of vasoconstrictive (angiotensin II) and blood clotting factors, hyperhomocysteinemia and inflammatory factors are frequently present [7-9]. Cigarette smoking, male gender, family history and lack of physical activity are also risk factors for CVD in CKD [10,11]. The greater the renal dysfunction, the greater the cardiovascular (CV) risk [11]. Even mild CKD was associated with higher mortality, more frequent hospitalization and the necessity of revascularization after primary coronary artery bypass graft (CABG) surgery during 7-year follow-up [12]. The study by Elsayedi *et al.*[13] revealed that individuals with CVD and abnormal renal function at baseline are at the greatest risk of rapid progression towards renal failure. Friedman *et al.*[14] suggested that common risk factors in chronic coronary artery disease (CAD) and CKD as well as atherosclerosis in the renal microcirculation have the greatest impact on the aforementioned phenomena. These studies underline the need for the detection and treatment of CKD in its early stages.

Cardiac arrhythmias are commonly associated with both heart disease and non-cardiovascular diseases, e.g. CKD [15]. In most patients cardiac arrhythmias are not life-threatening, but in the form of malignant ventricular arrhythmia it can lead to sudden cardiac death (SCD) [15]. In recent years, the opinions concerning risk assessment and the treatment of patients with dangerous ventricular arrhythmias have changed. The most significant change refers to the withdrawal of antiarrhythmic drug therapy, which itself may increase the risk of SCD [15]. Currently, the greatest attention is paid to the removal of the cause of arrhythmia and the use of non-pharmacological treatments, such as electrical ablation and the implantation of an automatic cardioverter defibrillator (implantable cardioverter defibrillator, ICD) [15,16].

The current review aims to present the current knowledge on pathogenesis and prevention of SCD in patients with CKD.

## Search strategy

We performed a search of the electronic databases [MEDLINE (1966 – June 2012), EMBASE and SCOPUS (1965 – June 2012), DARE (1966 – 2012)]. Additionally, abstracts from national and international cardiovascular meetings were studied. Where necessary, the relevant authors of these studies were contacted to obtain further data. The main data search terms were: arrhythmias, chronic kidney disease, dialysis, hemodialysis, prevention, renal failure, renal replacement therapy, sudden cardiac death, sudden death, therapy, treatment.

## Review

### Cardiac arrhythmias and SCD in patients with CKD

Cardiovascular deaths account for about 40% of all deaths of patients with chronic renal failure, particularly those on dialysis [17]. Hypertension which leads to the occurrence of left ventricular hypertrophy (LVH), heart failure (HF) and CAD or coexists with them plays the main role in the development of serious ventricular arrhythmias [18,19]. LVH essentially increases the risk of sudden cardiac death [20]. Half of patients with significant LVH develop symptoms of HF [13,14]. In the group of patients with end-stage renal disease (ESRD) and with hypertrophy (left ventricular mass index [LVMI] > 125 g/m^2^) 5-year survival was 20%, and 50% when the LVMI was <125 g/m^2^[13,14]. This is mainly due to QT prolongation and the increase in its dispersion. QT prolongation is typical for dialysis patients [13,14]. According to Herzog’s study [21] SCD might be responsible for as many as 60% of deaths in patients undergoing dialysis. Another study of 4,120 deaths during the 2-year follow-up of 12,833 hemodialysis patients revealed that the greatest percentage of all deaths (27%) was caused by sudden cardiac arrest, while other CV conditions accounted for 20% of all deaths [15,22]. In the another study by Herzog *et al.*[23], deaths from sudden cardiac arrest or arrhythmia accounted for approximately 25% of all-cause deaths. The study by Pun *et al.*[24] demonstrated an increased rate of SCD with increasing severity of CKD. Each 10 ml/min/1.73 m^2^ decline in eGFR was associated with increased risk of SCD (hazard ratio [HR] = 1.11; 95% CI 1.06–1.17, *p* < 0.001) [24].

According to studies the frequency of SCD increases with the time of dialysis treatment and with the time that passed from their previous dialysis session and it is highest among individuals with diabetes [25-27]. It was observed that the frequency of deaths of HD patients was higher than the expected ratio in the 12 h period from the beginning of dialysis treatment and on the last day of the dialysis-free weekend interval. The rate of cardiac arrest among patients with diabetes who were undergoing dialysis was 110 events per 1,000 patient-years at year one and rose to 208 events per 1,000 patient-years at year four [23,25]. In the already mentioned study by Herzog *et al.*[12] the survival rates after cardiac arrest were 32% at 30 days and 17% at 1 year after dialysis and dropped to 13% at 1 year in patients with diabetes.

Among other risk factors for SCD it is worth mentioning the hospitalization within 30 days after dialysis and a decrease of 30 mm Hg in systolic blood pressure (SBP) during hemodialysis [27]. Moreover, patients with ESRD and diabetes are at higher risk of sudden death than non-diabetic ESRD patients [28].

Previous studies have noted a number of factors occurring with HD that could lead to cardiac arrhythmias [15]. Patients with CKD undergoing hemodialysis are at high risk of ventricular arrhythmia and SCD since changes associated with renal failure and hemodialysis-related disorders overlap. It was revealed that ventricular arrhythmia is present in 90% and supraventricular arrhythmia in 80% of dialysis patients [15]. Cardiac arrhythmias are exacerbated by dialysis and several hours after it [15]. During dialysis, many changes occur that favor electrical instability, including disturbances in serum electrolytes, variations in various metabolite concentrations, acid–base balance alterations, the decrease in circulating blood volume, sympathetic overactivity as well as inflammation and possibly iron deposition [15,21,26]. Too low concentration of potassium in the dialysate, diabetes mellitus, advanced age and dialysis performed after the weekend break are important factors that increase the risk of SCD [26,29]. The study by Karnik *et al.*[26] showed that patients who died in the first 12 h interval had a lower mean serum potassium level in the prior monthly tests than those dying in the 60–72 h interval. Moreover, iron overload in ESRD increases the risk of SCD due to QT prolongation [29].

Concentric hypertrophy leads to the reduced susceptibility of the left ventricle [14,19]. In this situation, the ventricular filling pressure increases so that the ventricle could be filled sufficiently to ensure adequate cardiac output. The rise in filling pressure results in the appearance of symptoms of pulmonary congestion. On the other hand, the greater sensitivity of the hypertrophic ventricle results in its higher susceptibility to the occurrence of dialysis hypotension [30]. All these conditions favor the occurrence of serious ventricular arrhythmias and SCD. LVH increasing the risk of sudden death is associated with prolongation of the corrected QT interval or with increasing arrhythmogenesis [29].

According to studies also prolonged QT interval, QTc, and *torsade de pointes* occurring in CKD and ESRD can be a cause of sudden cardiac death [31]. Prolonged QTc in these patients results from inhomogeneity of myocardial depolarization and repolarization following LVH as well as intercardiomyocytic fibrosis [31,32]. Moreover, in patients with greater increases in QTc intervals after hemodialysis, baseline plasma calcium levels are higher and lower after hemodialysis, which suggests that QTc prolongation may be a result of calcium hemostasis abnormalities and may induce or predispose to sudden death [33]. A retrospective cohort study conducted on patients on dialysis QTc interval dispersion which occurred for longer than 74 ms was an independent predictor of all-cause mortality, CV mortality and arrhythmia-related mortality [34].

In patients with CKD inflammation can be also a common trigger of sudden cardiac death as a result of either premature atherosclerosis, and cytokine-induced plaque instability or by a direct effect on the myocardium and the electrical conduction system [35-37]. In this group of patients the level of toxins, proinflammatory cytokines, inflammatory mediators and reactive oxygen species (ROS) increases as renal function deteriorates [36-39]. Elevated levels of inflammatory mediators, the accumulation of asymmetric dimethylarginine, high levels of homocysteine as well as raised levels of calcification promoters (e.g. osteopontin), diminished levels of calcification inhibitors (e.g. fetuin-a) and abnormal calcium–phosphate metabolism are associated with atherosclerosis, atherothrombotic events and incident CV mortality in patients with CKD and those undergoing hemodialysis [40-43].

Myocardial ischemia is the most common cause of malignant ventricular arrhythmias in patients with CKD. CAD probably causes arrhythmogenesis in patients on hemodialysis since severe coronary stenosis is associated with the induction and lengthy persistence of ventricular arrhythmias during and after hemodialysis [15,44,45]. Arrhythmia also results from fibrosis and cardiac hypertrophy. Additional contractions and other changes in heart rate are its direct triggers. Ischemia, fluid and electrolyte disorders, autonomic neuropathy, anemia, ion disorders caused by the use of diuretics and valvular calcification are the modifying factors, which sometimes also become the triggering ones [15,21,26,46,47].

Ischemia is probably the most common factor which modifies the risk or elicits arrhythmia, but the role of changes in calcium-phosphate metabolism resulting in heart valve calcification should also be emphasized. These changes affect the efficiency of the heart and promote the occurrence of arrhythmias [48,49].

### The identification of patients at risk of SCD

The stratification of SCD risk in patients with CKD/renal failure has been poorly studied so far. Many studies have shown that elevated levels of troponin T (TnT), creatine kinase-MB (CK-MB) fraction or C-reactive protein (CRP) are associated with increased risk of death from CV causes. However, it is not known whether these markers also indicate the risk of sudden cardiac death [4]. The following risk factors for SCD in patients on dialysis are the most commonly mentioned: (1) demographic factors: male gender, black race, (2) data from family history: positive family history, smoking, low physical activity, (3) cardiovascular disease: hypertension, left ventricular hypertrophy (LVH), myocardial infarction (MI), angina pectoris, and (4) other diseases, such as: diabetes, hyperlipidemia, anemia, hyperparathyroidism, metabolic acidosis [4,24,35].

Patients at high risk of SCD can be also identified on the basis of markers of coronary ischemia such as ischemia modified albumin (IMA) or cardiac troponin [50]. Ischemia modified troponin is a novel biomarker which predicted all-cause mortality in ESRD patients with a sensitivity and specificity of 76% and 74%, respectively [50]. Elevated cardiac troponin (≥0.06 μg/l) predicted mortality with a sensitivity of 75% and a specificity of 72% [50]. Cardiac mortality risk was 7-fold higher in patients with elevated levels of both IMA and cardiac troponin (OR 7.12, 95%CI 4.14–10.12, *p* = 0.005) [50]. According to studies also severely impaired myocardial fatty acid metabolism due to recurrent myocardial ischemia may indicate those patients on dialysis who are at high risk of SCD [51].

Other small studies have shown that heart rate variability analysis and the assessment of baroreceptor sensitivity may also have predictive value. Impaired baroreflex effectiveness and sensitivity and obstructive sleep apnea may increase the risk of sudden death [52]. In the study of Johansson *et al.*[52] baroreflex sensitivity was reduced by 51% and the baroreflex effectiveness index by 49% in patients with hypertension and stage 4 or 5 CKD in comparison to age-matched healthy controls. According to another Johansson *et al.*[53] study, reduced baroreflex sensitivity was an independent predictor of SCD. The results of the German Diabetes Dialysis Study (4D) study demonstrated that 40% of people who died as a result of sudden cardiac arrest were found dead in bed in the morning, which according to the investigators might have been related to obstructive sleep apnea [54]. However, there were no measures or objective data available to support this conclusion. Moreover, this study revealed that low baseline homoarginine concentration was a strong risk factor for SCD and HF death during 4-year follow-up [55]. Another analysis of 4D data showed that poor glycemic control and vitamin D deficiency were also strongly associated with the incidence of SCD in dialysis patients [56]. Despite the progress of technology and numerous studies, current knowledge concerning the evaluation of risk SCD factors is still too limited to sufficiently explain the excess rate of such deaths in dialysis patients [55].

### Antiarrhythmic management in CKD patients

Antiarrhythmic therapy is much more difficult in patients with CKD, but the general principles are similar to those in patients with normal renal function. First of all, the cause of arrhythmias should be found and eliminated. In patients with CKD it is mainly based on very rigorous control of water and electrolyte balance. Antiarrhythmic drugs can be used in this group of patients but with limited effectiveness. The choice of therapy is limited due to the altered pharmacokinetics of many drugs resulting from renal failure, neurotoxicity of certain drugs and their complex interactions [57]. Beta-blockers should be the drugs of choice because of their lower risk of proarrhythmia [58,59]. The Multicenter Automatic Defibrillator Implantation Trial-II (MADIT II) study demonstrated that the use of these drugs was associated with a significant reduction in SCD risk in patients with CKD [60]. In another randomized controlled trial, carvedilol therapy reduced morbidity, all-cause and cardiovascular mortality (by 29.3%) in dialysis patients with ESRD and dilated cardiomyopathy [61]. The large (43,200 hemodialysis patients) retrospective study by Pun *et al.* revealed that beta-blockers were more frequently prescribed to patients who survived than to those who died from SCD (53% *vs* 40%, or 0.59, 95% CI 0.43–0.80, *p* = 0.0007) [62]. Moreover, according to this study beta-blockers were associated with a significantly lower risk of death at 24 h and 6 months after cardiac arrest [62].

Amiodarone seems to be the most effective antiarrhythmic drug used in the treatment of ventricular arrhythmia and atrial fibrillation. However, its numerous non-cardiac actions should be kept in mind [63]. In dialysis patients the reduction of SCD risk was achieved through the introduction of bicarbonate dialysis, modification of dialysis fluid composition (potassium and calcium concentration) depending on the clinical situation, rapid restoration of water-electrolyte and acid–base balance as well as monitoring of heart rhythm [64]. However, it is common knowledge that the implantation of automatic cardioverter-defibrillators (ICD) both as a primary and secondary prevention is the only effective way of preventing SCD [57]. Though ICD implantation decreases the risk of SCD, there are hardly any trials concerning the use of ICD in patients with advanced renal insufficiency [14]. The ICD is a device that works symptomatically and thus it does not decrease the risk of arrhythmic events, in contrast to coronary revascularization, optimal pharmacotherapy or cardiac resynchronization in HF [65,66]. Published data (MADIT II study) show that ICD implantation contributed to reduction in total mortality in primary and secondary prevention, and reduction in mortality from arrhythmia causes in a population of patients after myocardial infarction [67]. On the basis of the results of the aforementioned studies, guidelines considering the indications for ICD implantation were defined [68]. According to them, an ICD should be implanted as the secondary prevention in patients who survived cardiac arrest in the course of malignant ventricular arrhythmias unless arrhythmia occurred within the first 24-48 h after fresh myocardial infarction (class I of recommendation, level A of evidence). During the first 24-48 h ventricular arrhythmia is a symptom of acute ischemia, and treatment should refer to immediate and optimal coronary revascularization [68]. In the primary prevention of SCD, ICD should be implanted within 40 days after MI in patients with the EF 30-40%, in NYHA class II-III, receiving optimal pharmacological treatment and with predicted survival in good functional condition for at least one year (class I of recommendation, level A of evidence) [68].

The analysis of these recommendations reveals the existence of the so-called ‘grey area’ between 48 h and 40 days after MI. This early post-myocardial period raises the most controversy concerning SCD risk stratification and the possible implantation of ICD in primary prevention of SCD [69]. It is known that the risk of SCD is highest in that period. In the Valsartan in Acute Myocardial Infarction Trial (VALIANT) [70], 19% of patients died suddenly or were resuscitated within 30 days after myocardial infarction and 70% of all SCD occurred during the first year after MI. Although ICD implantation reduced the number of arrhythmic deaths, patients with implanted ICD died more often due to non-arrhythmic causes (non-SCD) [68-70].

On the basis of the aforementioned studies it can be concluded that while the ICD effectively interrupts ventricular arrhythmias in a population of patients who would suddenly die from arrhythmic causes without this device, it also contributes to the increased number of non-arrhythmic deaths. It was hypothesized that high energy therapies might have an adverse impact on the occurrence or exacerbation of HF in patients after MI [68,71]. The MADIT II study demonstrated that adequate ICD cardioversion/defibrillation increases threefold and inadequate defibrillation increases twofold the overall mortality [67]. This indicates high importance of correct and optimal programming of implanted devices in order to minimize the frequency of inappropriate ICD therapy. It is also known that the benefits of the implanted ICD increase with time after MI. The MADIT II study revealed a reduction in mortality and the greatest benefit from ICD implantation after over 18 months after myocardial infarction [67,69].

The study by Dasgupta *et al.*[72] conducted on 41 patients with ESRD and 123 controls demonstrated high complication rates for cardiac rhythm management devices (permanent pacemakers or implantable cardioverter-defibrillators) in this group of patients. Pneumothorax requiring a chest tube, pocket infection requiring device extraction, or thrombosis occurred in 29% of ESRD patients versus 5% of controls (*p* < 0.001) while minor complications occurred in 17% of ESRD patients versus 6% of controls (*p* < 0.03). However, no fatal complications were seen in either group [72]. A retrospective study of patients with ICD implanted due to primary or secondary indications revealed that renal insufficiency was a strong predictor of appropriate ICD shocks [73]. One-year incidence of appropriate ICD shock was 37.5% for patients on dialysis and 10.7% for those not on dialysis (*p* < 0.0001) [73].

The study of patients with or without CKD who received an ICD for primary prevention of sudden cardiac death demonstrated that 1-year survival of patients with CKD was lower (61.2%) than that of patients without CKD (96.3%) (*p* < 0.00001) [74]. In this study CKD was the most significant independent predictor of mortality (HR 10.5, 95%CI 4.8–23.1, *p* < 0.00001) [74]. The study of Korantzopoulos *et al.*[75], which demonstrated increased mortality in patients who receive ICD therapy, suggested that patients with advanced renal insufficiency could be less responsive to ICD therapy, probably due to higher defibrillation thresholds. Until the reasons for that phenomenon are explained it seems rational that patients with CKD and implanted ICD should be more frequently tested using the technique of programmed ventricular stimulation than patients with normal renal function [75]. This will help to determine the threshold of effective defibrillation. It is also possible that the incidence of infectious complications related to ICD implantation may be greater in this group. These and other factors are responsible for the lower frequency of ICD implantation in dialysis patients who survived cardiac arrest [75].

However, according to Herzog *et al.*[76] ICD implantation in cardiac arrest survivors on dialysis is associated with greater survival. They observed that estimated 1-, 2-, 3-, 4-, and 5-year survival after day 30 of admission in the ICD group were higher (71%, 53%, 36%, 25%, and 22%, respectively) in comparison to the no-ICD group (49%, 33%, 23%, 16%, and 12%) (*p* < 0.0001). In this study ICD implantation was associated with a 42% reduction in death risk [76].

It should be kept in mind that not all arrhythmic events are interrupted by the ICD, as it is in the case of e.g. pulseless electrical activity after ICD testing, permanent ventricular tachycardia or acute HF after defibrillation. Moreover, not every SCD results from arrhythmic causes or even CV-related ones. SCD (defined as sudden death occurring within one hour of the beginning of clinical symptoms) may well be the result of pulmonary embolism, aortic aneurysm dissection, stroke, etc. [68,77]. According to publications, worse survival and higher incidence of SCD were observed in patients with both ICD and CKD compared to patients with normal renal function [68,77-80].

According to the study by Cuculic *et al.*[14,74] in patients receiving an ICD for primary prevention of SCD, CKD significantly reduced long-term survival, which may limit the benefits of ICD therapy in this population. When making the decision concerning implantation of a ICD for primary prevention of sudden cardiac death in patients with CKD, the patient’s age and the stage of kidney disease should be taken into account [78]. According to this study ICDs reduce mortality in patients with stage 1 and 2 CKD. The benefits of ICD implantation are less notable in patients with stage 3–5 CKD [78]. These results could be explained by a higher procedural risk and complications in addition to decreased life expectancy in patients with advanced CKD [14,78]. Moreover, benefits of ICD implantation are age-dependent. Cardioverter-defibrillator implantation seems to be preferential in patients aged <80 years with GFR 30–59 ml/min/1.73 m^2^, aged <75 years with GFR 15–29 ml/min/1.73 m^2^ and aged <65 years with GFR <15 ml/min/1.73 m^2^[78].

A prospective study of the effectiveness of automatic cardioverter-defibrillators in a population of CKD patients is particularly necessary due to the very high incidence of sudden death in these patients. The role of drugs/invasive therapies in the prevention and treatment of SCD in CKD patients are briefly summarized in Table 1.

**Table 1:** The role of drugs/invasive therapies in the prevention and treatment of SCD in CKD patients

**DRUG/DEVICE**	**POPULATION**	**TYPE OF STUDY**	**NO. OF PTS.**	**RESULTS**	**LEVEL OF SIGNIFICANCE**	**REF.**
**BETA-BLOCKERS**	CKD (eGFR of <75 ml/min/1.73 m^2^) with LV dysfunction	Post-hoc analysis	1232	MADIT II study. Reduced risk of arrhythmic mortality in HF patients with mild renal insufficiency.	HR 0.61. 95%CI; 0.38-0.99.	[60]
**BETA-BLOCKERS**	HD patients who underwent cardiac arrest	Nested case–control cohort study	729	Significantly reduced risk of death with beta-blockers* at 24 h** at the 6-month time points***. Strongly predictive for survival.	* OR 0.32; 95%CI 0.17-0.61 ** *p* = 0.005 *** *p* < 0.001	[62]
**CARVEDILOL**	Dialysis patients with dilated cardiomyopathy	Prospective, randomized, placebo controlled	114	Reduced morbidity* and mortality** in dialysis patients with dilated cardiomyopathy. Carvedilol use is recommended in all dialysis patients with chronic HF.	* *p* < 0.00001 ** *p* < 0.01	[61]
**ICD**	ESRD patients and controls who had permanent pacemaker or ICD	Observational study	41 with ESRD; 123 controls	Patients with renal insufficiency are more prone to develop ventricular tachyarrhythmia and receive appropriate ICD shocks^2^.	* p < 0.001	[72]
**ICD**	Patients with renal insufficiency and ICD	Prospective	230	Renal insufficiency is a strong predictor of ICD shocks and antitachycardia pacing.	*p = 0.02*	[73]
**ICD**	CKD patients who underwent ICD	Retrospective study	35 with CKD (total 229)	In patients receiving an ICD for primary prevention of sudden death, CKD significantly reduced long-term survival.	HR 10.5; 95% CI 4.8-23.1; *p = 0.0001*	[74]
**ICD**	CKD with ICD^*^ or eGFR < 60 mL/min/1.73 m^2**^ with ICD	Meta-analysis	3010	CKD is associated with increased mortality in patients who receive ICD therapy.	HR = 3.44, 95% CI 2.82-4.21, p < 0.001^*^HR = 3.06, 95% CI 2.31-4.04, p < 0.001^**^	[75]
**ICD**	Dialysis patients with ventricular fibrillation/cardiac arrest and ICD	Retrospective cohort observational study	460 (ESRD + ICD), 5582 without ICD	Estimated 1 to 5-year survival after ICD implantation was better than in the non-ICD group*. ICD implantation was independently associated with a 42% reduction in death risk**	* *p* < 0.0001 ** RR 0.58, 95%CI 0.50-0.66	[76]
**ICD**	CKD patients with ICD	Prospective study	-	The beneficial effect of an ICD for primary prevention of SCD in CKD patients depends primarily on the patient’s age and stage of renal disease. ICD implantation in stages I-II reduces mortality, but in more advanced stages, advantages are less pronounced and are age-dependent due to higher procedural risk and decreased life expectancy.	-	[78]

ABBREVIATIONS: *SCD*, sudden cardiac death; *CKD*, chronic kidney disease; *MADIT II*, Multicenter Automatic Defibrillator Implantation Trial-II; *HR*, hazard risk; *OR*, odds ratio; *HF*, heart failure; *VALIANT*, Valsartan in Acute Myocardial Infarction Trial; *ICD*, implanted cardioverter-defibrillator; *ESRD*, end-stage renal disease, *RR*, risk reduction.

### The role of electrostimulation

Pathological bradyarrhythmias (<50/min) resulting from sinus node or AV conduction dysfunction are responsible for 15-20% of SCDs [67,68]. Therefore, the implantation of classic one- and two-electrode stimulators, as well as pacemaker systems, is extremely important in the prevention of SCD. Sinus node disease, known as sick sinus syndrome (SSS), involves the entire group of sino-atrial disorders, from sinus bradycardia to sinus inhibition or so-called bradycardia-tachycardia syndrome. Sinus node disease occurs much more frequently in elderly patients and it is believed to be a major reason for permanent pacemaker implantation in this group [67,68,81,82]. Sinus node degeneration progresses with age, which means the reduction of the number of nodal cells, and the increase in the content of connective tissue and fat mainly in the nodal area. Symptoms of this disease include decreased exercise tolerance and cognitive impairment (mainly in elderly patients) due to bradycardia and chronotropic insufficiency resulting from the inability to properly increase heart rate in response to exercise. Atrial tachyarrhythmia occurring after a sinus pause may increase the heart rate so strongly that it may lead to hypotension and loss of consciousness. Elderly people lose consciousness easily due to numerous other contributory factors, such as baroreceptor failure, polytherapy and changes in the cerebral vessels [67,68,81,82]. The determination of indications for pacemaker implantation in patients with sinus node disease should be preceded by a detailed differential diagnosis including cerebral circulation disorders, which can also be a cause of dizziness, balance impairment or loss of consciousness [82].

The selection of stimulation type in patients with sinus node disease should depend on the type of disorders and symptoms. CKD is not a contraindication for the implantation of dual-chamber pacing with the minimization of right ventricular stimulation. Such procedures are validated by the improvement of the quality of life resulting from the limitations of atrial fibrillation attacks, which are in elderly patients a common cause of significant cardiac decompensation. Moreover, in patients with sinus node disease, there is a risk of future atrioventricular conduction disorders [68,82].

### Implantation of cardiac resynchronization system

Cardiac resynchronization therapy (CRT) with or without ICD is used to treat advanced HF accompanied by inter- and intraventricular conduction abnormalities leading to significant desynchronization of chambers [83,84]. CRT is complementary to optimal drug therapy. The Comparison of Medical Therapy, Pacing and Defibrillation in Heart Failure (COMPANION) study compared the clinical effects of CRT-D or CRT-P system implantation [84,85]. The analysis of the results from this study revealed that only CRT-D system implantation was associated with a significant reduction in the risk of SCD. According to the 2010 guidelines concerning HF and cardiac resynchronization therapy, CRT should be implanted in the following patients: (1) with heart failure (NYHA class III or IV), EF <35%, QRS > 120 ms, sinus rhythm, treated with a stable pharmacological medical regimen. Furthermore, patients in NYHA class IV should not be hospitalized for HF during the month prior to device implantation and their expected survival should be longer than 6 months (I/A); (2) with heart failure in NYHA class II, EF <35%, QRS > 150 ms, sinus rhythm, treated with a stable pharmacological medical regimen (I/A) [86].

The implantation of CRT systems is more and more common due to both the extension of indications as well as greater availability of this method. The effectiveness of resynchronization was not assessed in patients with CKD. However, it seems highly possible that this method can be effective also in patients with CKD and symptoms of systolic HF and contraction asynchronism.

## Conclusions

Over the last decade, numerous studies have confirmed the strong association between CKD and an increased risk of cardiovascular diseases. The decision concerning the implantation of a pacing system in patients with CKD should be made on the basis of careful individual assessment of the patient. Treatment of arrhythmias in patients with CKD is more difficult than in patients with preserved renal function. The control and correction of electrolyte disturbances, the treatment of cardiovascular disease, and the implantation of ICD in patients at highest risk are very important in this group of patients [87-91].

## Competing interests

The authors declare that they have no competing interests.

## Authors’ contributions

BFS and AG participated in the design of the study, made the literature search and drafted the manuscript. MB participated in the design of the study and prepared the final version of the manuscript as well as responses to reviewers. DK, JM and JR participated in the coordination and helped to draft the manuscript. All authors read and approved the final manuscript.

## Pre-publication history

The pre-publication history for this paper can be accessed here:

http://www.biomedcentral.com/1471-2369/13/162/prepub
